# Supplementary material for: Conditional chemoconnectomics (cCCTomics) as a strategy for efficient and conditional targeting of chemical transmission
Source: eLife. 2024 Apr 30;12:RP91927. doi: 10.7554/eLife.91927 (PMC11060718; doi:10.7554/eLife.91927)
Supplement: Figure 3—source data 3. [file elife-91927-fig3-data3.zip › Figure 3 source data3 part 2.zip!/Figure 3-Source data 3-part2/1062-13-CRISPResso_Report_jt28fhr9.zip!/CRISPResso_on_1062_14/9.gcaggaaatgagctacctac.Alleles_frequency_table_around_sgRNA_gcaggaaatgagctacctac.pdf]

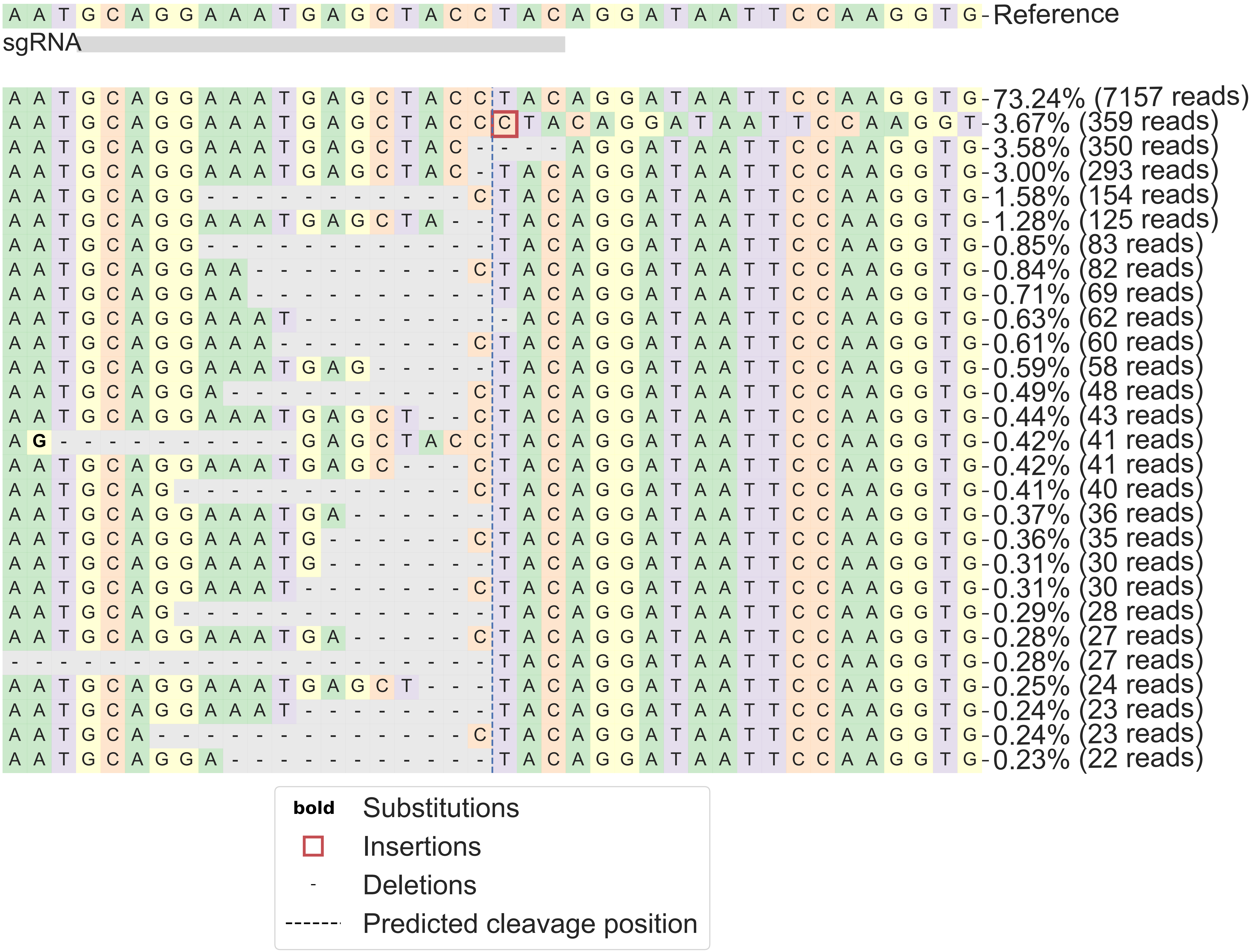
AATGCAGGAAATGAGCTACCTACAGGATAATTCCAAGGTG Reference
sgRNA
AATGCAGGAAATGAGCTACCTACAGGATAATTCCAAGGTG 73.24% (7157 reads)
AATGCAGGAAATGAGCTACCCTACAGGATAATTCCAAGGT 3.67% (359 reads)
AATGCAGGAAATGAGCTAC----AGGATAATTCCAAGGTG 3.58% (350 reads)
AATGCAGGAAATGAGCTAC-TACAGGATAATTCCAAGGTG 3.00% (293 reads)
AATGCAGG-----------CTACAGGATAATTCCAAGGTG 1.58% (154 reads)
AATGCAGGAAATGAGCTA--TACAGGATAATTCCAAGGTG 1.28% (125 reads)
AATGCAGG------------TACAGGATAATTCCAAGGTG 0.85% (83 reads)
AATGCAGGAA---------CTACAGGATAATTCCAAGGTG 0.84% (82 reads)
AATGCAGGAA----------TACAGGATAATTCCAAGGTG 0.71% (69 reads)
AATGCAGGAAAT---------ACAGGATAATTCCAAGGTG 0.63% (62 reads)
AATGCAGGAAA--------CTACAGGATAATTCCAAGGTG 0.61% (60 reads)
AATGCAGGAAATGAG-----TACAGGATAATTCCAAGGTG 0.59% (58 reads)
AATGCAGGA----------CTACAGGATAATTCCAAGGTG 0.49% (48 reads)
AATGCAGGAAATGAGCT--CTACAGGATAATTCCAAGGTG 0.44% (43 reads)
AG----------GAGCTACCTACAGGATAATTCCAAGGTG 0.42% (41 reads)
AATGCAGGAAATGAGC---CTACAGGATAATTCCAAGGTG 0.42% (41 reads)
AATGCAG------------CTACAGGATAATTCCAAGGTG 0.41% (40 reads)
AATGCAGGAAATGA------TACAGGATAATTCCAAGGTG 0.37% (36 reads)
AATGCAGGAAATG------CTACAGGATAATTCCAAGGTG 0.36% (35 reads)
AATGCAGGAAATG-------TACAGGATAATTCCAAGGTG 0.31% (30 reads)
AATGCAGGAAAT-------CTACAGGATAATTCCAAGGTG 0.31% (30 reads)
AATGCAG-------------TACAGGATAATTCCAAGGTG 0.29% (28 reads)
AATGCAGGAAATGA-----CTACAGGATAATTCCAAGGTG 0.28% (27 reads)
--------------------TACAGGATAATTCCAAGGTG 0.28% (27 reads)
AATGCAGGAAATGAGCT---TACAGGATAATTCCAAGGTG 0.25% (24 reads)
AATGCAGGAAAT--------TACAGGATAATTCCAAGGTG 0.24% (23 reads)
AATGCA-------------CTACAGGATAATTCCAAGGTG 0.24% (23 reads)
AATGCAGGA-----------TACAGGATAATTCCAAGGTG 0.23% (22 reads)
bold Substitutions
Insertions
- Deletions
------- Predicted cleavage position
